# Minimum detectable and minimal clinically important changes for pain in patients with nonspecific neck pain

**DOI:** 10.1186/1471-2474-9-43

**Published:** 2008-04-10

**Authors:** Francisco M Kovacs, Víctor Abraira, Ana Royuela, Josep Corcoll, Luis Alegre, Miquel Tomás, María Antonia Mir, Alejandra Cano, Alfonso Muriel, Javier Zamora, María Teresa Gil del Real, Mario Gestoso, Nicole Mufraggi, The Spanish Back Pain Research Network

**Affiliations:** 1 Departamento Científico, Fundación Kovacs, Paseo de Mallorca 36, E-07012, Palma de Mallorca, Spain; 2 Unidad de Bioestadística Clínica, Hospital Ramón y Cajal, Carretera de Colmenar, km 9.100 E-28034 Madrid, Spain; 3 Centro de Salud de Tramuntana Esporlas – Mallorca, C/Coarter 24, E-07190 Esporlas, Mallorca, Spain; 4 Servei de Planificació i Coordinació Asistencial, Servei de Salut de les Illes Balears (Ib-Salut), C/Reina Esclaramunda 9, E-07003 Palma de Mallorca, Spain; 5 Servei d'Atenció Primària i 061, Servei de Salut de les Illes Balears (Ib-Salut), C/Fábrica 56, E-07013 Palma de Mallorca, Spain; 6 Centro de Salud de Inca, C/Castell de Bellver s/n E-07300 Inca, Mallorca, Spain; 7 CIBER Epidemilogía y Salud Pública (CIBERESP), Spain

## Abstract

**Background:**

The minimal detectable change (MDC) and the minimal clinically important changes (MCIC) have been explored for nonspecific low back pain patients and are similar across different cultural settings. No data on MDC and MCIC for pain severity are available for neck pain patients. The objectives of this study were to estimate MDC and MCIC for pain severity in subacute and chronic neck pain (NP) patients, to assess if MDC and MCIC values are influenced by baseline values and to explore if they are different in the subset of patients reporting referred pain, and in subacute versus chronic patients.

**Methods:**

Subacute and chronic patients treated in routine clinical practice of the Spanish National Health Service for neck pain, with or without pain referred to the arm, and a pain severity ≥ 3 points on a pain intensity number rating scale (PI-NRS), were included in this study. Patients' own "global perceived effect" over a 3 month period was used as the external criterion. The minimal detectable change (MDC) was estimated by means of the standard error of measurement in patients who self-assess as unchanged. MCIC were estimated by the mean value of change score in patients who self-assess as improved (mean change score, MCS), and by the optimal cutoff point in receiver operating characteristics curves (ROC). The effect on MDC and MCIC of initial scores, duration of pain, and existence of referred pain were assessed.

**Results:**

658 patients were included, 487 of them with referred pain. MDC was 4.0 PI-NRS points for neck pain in the entire sample, 4.2 for neck pain in patients who also had referred pain, and 6.2 for referred pain. MCS was 4.1 and ROC was 1.5 for referred and for neck pain, both in the entire sample and in patients who also complained of referred pain. ROC was lower (0.5 PI-NRS points) for subacute than for chronic patients (1.5 points). MCS was higher for patients with more intense baseline pain, ranging from 2.4 to 4.9 PI-NRS for neck pain and from 2.4 to 5.3 for referred pain.

**Conclusion:**

In general, improvements ≤ 1.5 PI-NRS points could be seen as irrelevant. Above that value, the cutoff point for clinical relevance depends on the methods used to estimate MCIC and on the patient's baseline severity of pain. MDC and MCIC values in neck pain patients are similar to those for low back pain and other painful conditions.

## Background

Minimal detectable change (MDC) is defined as the minimal change that falls outside the measurement error in the score of an instrument used to measure a symptom. Minimal clinicall important change (MCIC) is defined as the minimal change in the score that is meaningful for patients [1-12]. Different approaches can be used to determine MCIC. One is to estimate the mean change in score in patients who actually report to have improved (referred to as "mean change score", or MCS). Another approach is to use receiver operating characteristics curves (ROC) to define the cutoff point that best discriminates between patients reporting or denying any improvement. MDC, MCS and ROC reflect different constructs, and the methods for assessing each one of them are also different. These methods are further described in the "Methods" section of this paper, and they lead to different values [8,9,12].

It is useful to define MDC and MCIC for three main reasons: a) to calculate the sample size of studies aiming to assess the effectiveness or cost/effectiveness of interventions, b) to interpret the clinical relevance of results in studies on the effectiveness of treatments, and c) to help clinicians select among treatments with slight differences in their size effects, especially if they differ in their safety profiles, by allowing them to anticipate the clinical meaningfulness of the expected differences in their effects.

MDC and MCIC for low back pain patients have been estimated in different cultural settings, with consistent results [1-9]. In general, improvements of pain severity ≤ 1.5 points on a pain intensity numerical rating scale (PI-NRS) could be seen as clinically irrelevant [6-9]. Above that value, the cutoff point for "clinical relevance" depends on patients' baseline pain severity and on the method used for estimating it [8,9]. Results are similar in LBP patients with and without referred pain [9]. Values for MDC and MCIC are different across acute and chronic LBP patients, [8] but they are stable in subacute and chronic patients [9].

In patients with nonspecific neck pain (NP), MDC and MCIC have been explored for disability and quality of life [13-15]. MDC and MCIC for pain severity have also been recently explored [16]. However, MDC and MCIC for pain were only explored in a small sample of patients participating in a randomized controlled trial, it is unknown whether or not they had referred pain, and approximately 75% of them were acute patients [16], while subacute and chronic patients represent the major part of the social, clinical and economical burden associated with spinal disorders [17]. MCIC for neck pain might be different between acute and chronic patients, between patients with and without referred pain, and between patients seen in routine clinical practice and those included in a randomized controlled trial in which expectations and other unspecific effects (such as Hawthorne or placebo) might also influence them.

Therefore, the primary objective of this study was to calculate the MDC and MCIC values for neck and referred pain severity in subacute and chronic NP patients treated in routine clinical practice. Additional objectives were: a) to explore if MDC and MCIC values for neck pain were different in the subset of patients who reported referred pain, b) to assess if MDC and MCIC values depended of baseline values, and c) to explore if they were different between subacute and chronic patients.

## Methods

### Setting

This study was performed in the Balearic Health Service (Ib-Salut). The Ib-Salut is a public organization that belongs to the Spanish National Health Service, in which universal, tax funded health care services are provided to every citizen. The Ib-Salut covers all of the inhabitants of the Balearic Islands, who were about 916,500 when this study started. All of the 515 primary care physicians working at the 49 primary care centers belonging to the Ib-Salut were invited to participate in this study. No incentives were offered to the physicians or patients to participate.

Data from patients recruited between January 1, 2004, and November 21, 2006, were included in this study. The study was approved by the Medical Ethics Committees of the participating institutions.

### Study population

The study population was defined as patients seeking care in the routine primary care practice of the Ib-Salut for subacute and chronic NP, with or without referred pain. Based on the available evidence for LBP patients, time limits to consider a patient as "subacute" and "chronic" were set at between 14 and 90 days, and longer than 90 days, respectively [18-20].

In accordance with the current treatment protocol for neck pain in routine practice in the Ib-Salut, subacute and chronic patients were referred to a specialized Unit, where patients with a pain severity ≥ 3 points on a pain intensity numerical rating scale (PI-NRS) [21], underwent a neuroreflexotherapy (NRT) intervention. This is a minimally invasive procedure that has proven to be safe, effective and cost/effective, and has been extensively described in the literature [22-27]. Data included in the current study derive from methods used for post-marketing surveillance of this technology in routine clinical practice [22].

Inclusion criteria were: seeking health care at any of the primary care centers belonging to the Ib-Salut, for neck pain (NP) lasting 14 or more days, either with or without pain referred to the arm or arms, reporting a pain severity ≥ 3 points on a pain intensity numerical rating scale (PI-NRS) [20], and undergoing a neuroreflexotherapy (NRT) intervention.

Exclusion criteria were [28]: data suggesting potential underlying diseases, (the current neck pain episode being the first one in a patient under age 20 or onset over 55 in which appropriate diagnostic procedures had not yet been performed before referral to the study, non-mechanical pain, widespread neurology (disseminated neurological findings), fever, weight loss, systemic unwellness, a history of: significant trauma, systemic steroids, osteoporosis, cancer, drug abuse, HIV), widespread (>1 nerve root) or progressive motor weakness in the arms, and patients' refusal to sign the study's informed consent.

### Outcome Assessment

The clinical condition of each patient was evaluated by the GPs at the primary care centers, who gathered data on duration of the current pain episode (in days); existence of referred pain (yes/no); diagnostic procedures performed in the previous 6 months, and current treatment. Patients were assessed on their first visit (baseline assessment) and three months later (12 week follow-up).

At each assessment, patients rated, on their own and without assistance, two separate PI-NRS for current neck and referred pain, ranging from "0" (no pain) to "10" (worst imaginable pain). NP-related disability was not assessed since no validated instrument for that purpose is available in Spanish.

### External criterion

At the 3 month follow-up, patients scored the change in their clinical status on the following scale: 1: Completely recovered, 2: improved, 3: unchanged, 4: worsened. Patients rated their clinical status on their own and without assistance.

Since patients graded the evolution of their own clinical status, this classification was considered as the external criterion for a change to be "clinically important".

### Analysis

The MDC and MCIC values in the PI-NRS for neck and referred pain were estimated for the follow-up period of 3 months.

The Minimal Detectable Change (MDC) was calculated as 1.96 * √2 * SEM. [8,12] The standard error of measurement (SEM) was estimated by taking the square root of the within subject variance (consisting of variance between measures plus the residual variance on a two-way ANOVA random effects model) of patients categorized as "unchanged" by external criterion. The 95% confidence interval was calculated using the chi-square distribution [29]. The MDC can be interpreted as the magnitude of change below which there is more than a 95% chance that no real change has occurred.

The following methods were used to estimate the MCIC [12]:

1. Mean Change Score (MCS): Mean change of PI-NRS in patients who scored "2" ("improved") on the external criterion. The changes of scores PI-NRS were calculated by subtracting the final values from the baseline values, so that positive scores correspond to improvement.

2. Optimal cutoff point (ROC): Considering the PI-NRS change as a diagnostic test for discriminating between improved and not improved patients, and the external criterion as a gold standard, a ROC curve was developed describing the performance of changes in the corresponding scale to detect improvement [30]. The optimal cutoff point was estimated by the point that maximizes the sum of specificity and sensitivity.

Data from all recruited patients (both with and without referred pain) were included in the main analysis, in which MDC and MCIC values for neck pain were calculated. In a subgroup analysis, only patients with referred pain at baseline were included, and MDC and MCIC values for neck and referred pain were calculated.

The effects of baseline scores and chronicity on MDC, MCS and ROC were estimated by defining subgroups. Values were estimated for patients with low baseline scores (lowest tertile) and high baseline scores (highest tertile). Values were also estimated for chronic and subacute patients, with the cut-off point for chronicity established at 90 days [1,30]. All statistical analyses were performed using SPSS for Windows, version 12.0.

## Results

In this study 678 patients were recruited and 20 (2.9%) were excluded because of refusal to sign the informed consent (15 cases), fever (3 cases) and patients feeling systematically unwell (2 cases). Therefore, 658 were included, and none were lost to follow-up. As seen in Table 1, in general they were middle aged working women, with intense NP. 487 (74.0%) also reported referred pain, and slightly over half were chronic. Twenty subjects (2.9%) were excluded from the analysis because of missing values corresponding to pain severity at baseline or discharge, or on the scale corresponding to their perceived effect.

**Table 1:** Baseline characteristics of all included patients.

Variable	All included patients (N = 658)	Patients with referred pain (N = 487)
Age; Mean (SD)	54.1 (14.6)	53.8 (14.2)
Gender; n (%)		
Male	148 (22.5)	94 (19.3)
Female	510 (77.5)	393 (80.7)
Work status; n (%)		
Active	606 (92.1)	453 (93.0)
Retired	52 (7.9)	34 (7.0)
Duration of present episode (days);		
Mean (SD)	541.7 (1274.9)	580.3 (1364.8)
Chronicity:		
Subacute (14–90 days)	290 (44.6)	202 (41.8)
Chronic (> 90 days)	361 (55.4)	281 (58.2)
Baseline neck pain (PI-NRS points);		
N	658	485
Mean (SD)	7.2 (2.0)	7.3 (2.0)
Baseline pain referred to the arm (PI-NRS points);		
N	--	487
Mean (SD)		6.6 (2.4)

PI-NRS: Pain intensity numerical rating scale

Three months later, 210 (31.9%) reported feeling completely recovered, 395 (60.0%) improved, 48 (7.3%) unchanged, and only 5 (0.8%) worse. Table 2 shows neck and referred pain scores at baseline and changes in scores 3 months later, for patients who reported to have "completely recovered", "improved", "not changed" or "worsened".

**Table 2:** Baseline values and final change of scores for neck pain and pain referred to the arm across different values for external criterion

Value	Baseline neck pain^1^	Evolution of neck pain^1^	Baseline referred pain^1,2,3^	Evolution of referred pain^1,2,3^
Completely Recovered				
N	210	210	142	137
Mean (SD)	6.7 (2.0)	6.1 (2.1)	6.4 (2.5)	6.0 (2.5)
Improved				
N	395	395	307	304
Mean (SD)	7.4 (2.0)	4.1 (2.3)	6.7 (2.4)	4.0 (2.7)
Unchanged				
N	48	48	35	35
Mean (SD)	7.2 (2.1)	0.8 (1.8)	7.0 (2.2)	1.6 (2.7)
Worsened				
N	5	5	3	3
Mean (SD)	7.8 (1.9)	-1.2 (1.6)	8.3 (0.6)	-0.7 (1.5)

1: Points on a pain intensity numerical rating scale. 2: Only for patients with referred pain at baseline. 3: Positive values in change correspond to improvement, and negative values to worsening.

Table 3 shows the MCIC values for neck pain estimated in all included patients, while Table 4 shows the MCIC values for both neck and referred pain only in patients who also reported referred pain at baseline.

**Table 3:** MDC, MCS and ROC values for neck pain in all patients included in the study (with and without referred pain), and differences depending on baseline pain severity and chronicity

Measurement of change	Value (all 658 patients)	Baseline pain severity (PI-NRS)		Chronicity	
		Lowest tertile* (< 6 points)	Highest tertile* (≥ 8 points)	Subacute (<90 days)	Chronic (≥ 90 days)

**Neck pain **(PI-NRS)					
MDC; Value (95% CI)**	4.0 (3.4–5.0)				
n	48	14	27	10	38
MCS (SD)	4.1 (2.3)	2.6 (1.4)	4.9 (2.3)	4.1 (2.3)	4.1 (2.2)
n	395	78	209	167	223
ROC curve					
Area (IC 95%)	0.92 (0.87–0.96)	0.94 (0.85–1.00)	0.95 (0.91–0.99)	0.93 (0.80–1.00)	0.91 (0.86–0.96)
Sensitivity	0.93	0.89	0.96	0.99	0.93
Specificity	0.83	0.93	0.80	0.90	0.81
ROC	1.5	1.5	1.5	0.5	1.5
N	658	164	321	290	361

PI-NRS: Pain intensity numerical rating scale.

MDC: Minimal detectable change. MCS: Mean change score. ROC: Optimal cutoff point on the ROC curve.

*: Lowest and highest tertiles do not represent the same number of patients because of repeated scores for pain severity.

1.2.1:** The low number of patients denying any change in this sample made it impossible to reliably estimate the potential effect of baseline pain severity and chronicity status on MDC values.

**Table 4:** MDC, MCS and ROC values for neck pain and referred pain only in patients with referred pain at baseline, and differences depending on baseline pain severity and chronicity

Measurement of change	Value (all 487 patients with referred pain at baseline)	Baseline pain severity (PI-NRS)		Chronicity	
		Lowest tertile* (< 6 points)	Highest tertile* (≥ 8 points)	Subacute (<90 days)	Chronic (≥ 90 days)

**Neck pain **(PI-NRS)					
MDC; Value (95% CI)**	4.2 (3.4–5.5)				
n	34	11	19	6	28
MCS (SD)	4.1 (2.3)	2.5 (1.2)	4.8 (2.4)	4.1 (2.4)	4.1 (2.3)
n	306	61	167	120	182
ROC curve					
Area (IC 95%)	0.91 (0.86–0.97)	0.92 (0.80–1.00)	0.93 (0.88–0.99)	1.00 (0.99–1.00)	0.89 (0.82–0.96)
Sensitivity	0.93	0.88	0.87	0.99	0.93
Specificity	0.81	0.91	0.86	1.00	0.77
ROC	1.5	1.5	2.5	0.5	1.5
N	485	114	249	202	279
**Pain referred to the arm **(PI-NRS)					
MDC; Value (95% CI)**	6.2 (5.0–8.2)				
n	35	11	19	7	30
MCS (SD)	4.1 (2.7)	2.4 (1.7)	5.3 (3.0)	3.7 (3.4)	3.6 (3.2)
n	304	96	127	127	193
ROC curve					
Area (IC 95%)	0.81 (0.73–0.89)	0.80 (0.65–0.95)	0.86 (0.76–0.96)	0.89 (0.78–1.00)	0.76 (0.68–0.85)
Sensitivity	0.87	0.79	0.87	0.90	0.81
Specificity	0.71	0.82	0.77	0.86	0.71
ROC	1.5	1.5	2.5	0.5	1.5
N	479	159	201	214	293

PI-NRS: Pain intensity numerical rating scale

MDC: Minimal detectable change. MCS: Mean change score. ROC: Optimal cutoff point on the ROC curve.

*: Lowest and highest tertiles do not represent the same number of patients because of repeated scores for pain severity.

1.2.1:** The low number of patients denying any change in this sample made it impossible to reliably estimate the potential effect of baseline pain severity and chronicity status on MDC values.

As seen in those tables, MDC for neck pain is 4.0 PI-NRS points in the entire sample and it is 4.2 for patients who also complained from referred pain, and MDC for referred pain is 6.2 PI-NRS points. These values are not influenced by baseline pain severity or duration.

MCS for neck pain is 4.1 PI-NRS points, both in the entire sample and in patients who also had referred pain, and it is also 4.1 PI-NRS for referred pain. MCS remains constant across subacute and chronic patients, but it is higher for patients with more severe baseline pain. Depending on baseline severity, MCS values range from 2.5 to 4.9 PI-NRS points for neck pain, and from 2.4 to 5.3 for referred pain (Tables 3 and 4).

ROC for neck pain is 1.5 PI-NRS points, ranging from 1.5 to 2.5 depending on baseline pain severity, and from 0.5 in subacute patients to 1.5 in chronic ones. Those values for neck pain are identical in the entire sample and in patients who also had referred pain, and they are also identical for neck and referred pain (Tables 3 and 4).

## Discussion

Results from this study show that MCIC values are similar for neck and referred pain, and that MCIC for neck pain are also similar in subacute and chronic patients seen in routine clinical practice and in the subset of those subjects also reporting referred pain. In addition, these results suggest that improvement in pain below 1.5 PI-NRS could be seen as irrelevant, and that the cutoff point considering change as "clinically relevant" above that value depends on the method used to estimate MCIC and baseline severity of symptoms (Tables 3 and 4). All of those findings are consistent with those from previous studies on low back pain (LBP) patients [8,9]. It has been suggested that the MCS being larger in patients with a higher baseline pain severity is due to a smaller change potential in patients with lower baseline scores [8,9], or to patients with more severe pain needing to experience a greater improvement in order to feel that it is relevant [9].

In spite of differences in methods commented on in the Introduction section, present results are consistent with those from the only previous study in which MDC and ROC were explored for neck pain [16]. In that study, MDC for neck pain was 4.3 PI-NRS points, and ROC was 2.5 [16]. In the current study, those values were 4.0 and 1.5.

In fact, the size of MDC, MCS and ROC values in this study is similar to those found in LBP patients across studies performed with different samples in different cultural and geographical settings [6-9]. In the current study, participants' age and baseline pain severity for both local and referred pain were similar to those in previous studies with LBP patients, but 9% more women were included, the proportion of chronic patients was 8% less, and mean duration of the current pain episode was 153 days shorter (Table 1) [9]. In spite of those differences, in the current study, MDC values are 4.0 PI-NRS points for neck pain and 6.2 for referred pain (Tables 3 and 4), and corresponding values in low back pain patients are similar (3.5 and 5.4 points) [9]. MDC computation relies on the assumption of data being drawn from a normally distributed population. Although some of the subgroups defined by chronicity or pain severity were skewed, results for all of them are presented for completeness, and to permit comparisons with results from similar studies [8,9,16]. In this study, MCS and ROC values for neck pain are 4.1 and 1.5 PI-NRS points (Table 3), while corresponding values in low back pain patients were 4.4 and 1.5 PI-NRS [9]. Similarly, in this study MCS and ROC values for referred pain are 4.1 and 1.5 PI-NRS points (Table 4), while corresponding values for referred pain in low back pain patients are 4.3 and 2.5 PI-NRS points [9]. Range of MCS values depending on baseline pain severity are also consistent with those found in low back pain patients (Tables 3 and 4) [8,9].

In general, these results are also consistent with those from studies conducted on neck pain and on other painful conditions [10,11]. The consistency of those findings could be interpreted as contributing to the validity of the MDC and MCIC values deriving from the current study.

Results from this study show that ROC value for referred pain is 1.5 PI-NRS points (Table 4), while the mean change in PI-NRS score among patients denying any change in that variable is 1.6 (Table 2). It may seem conceptually paradoxical for ROC value to be lower than mean change in patients denying any improvement. In fact, this finding is due to the asymmetrical distribution of score changes in the patients denying any improvement in referred pain (Table 5) and, in practice, it does not affect the validity of ROC value found in this study.

**Table 5:** Frequency distribution [n (%)] of change in referred pain severity according to external criterion.

Change in PI-NRS^#^	**Patients Reporting Complete Recovery **(n = 137)	**Patients reporting improvement **(n = 304)	**Patients reporting no change **(n = 35)	**Patients reporting worsening **(n = 3)
10	13 (9,5)	12 (3,9)	0	0
9	11 (8,0)	8 (2,6)	1 (2,9)	0
8	18 (13,1)	21 (6,9)	1 (2,9)	0
7	18 (13,1)	20 (6,6)	1 (2,9)	0
6	17 (12,4)	26 (8,6)	1 (2,9)	0
5	22 (16,1)	35 (11,5)	0	0
4	9 (6,6)	37 (12,2)	4 (11,4)	0
3	16 (11,7)	51 (16,8)	1 (2,9)	0
2	8 (5,8)	42 (13,8)	2 (5,7)	0
1	4 (2,9)	30 (9,9)	9 (25,7)	1 (33,3)
0	1 (0,7)	13 (4,3)	12 (34,3)	0
-1	0	5 (1,6)	1 (2,9)	1 (33,3)
-2	0	4 (1,3)	2 (5,7)	1 (33,3)

# Difference between baseline and final assessment scores, in PI-NRS points. Positive values reflect improvement, and negative ones worsening.

The low number of patients in this sample denying any change made it impossible to reliably estimate the potential effect of baseline pain severity and chronicity status on MDC values (Tables 2, 3, 4). However, results from this study show that, although MDC and MCS are different concepts and the methods used to calculate their values are also different, their size for neck pain is very similar both in all the patients and in the subset of those complaining from referred pain. This finding is also consistent with results from previous studies, both in neck and low back pain patients [8,9,16]. It might be interpreted that the limit of what constitutes a "relevant" and a "detectable" change is similar whether it derives from the scores of patients who report improvement or deny it. However, as opposed to ROC, those calculations do not take into account false positives and false negatives.

Although it is up to researchers or clinicians to decide whether MCS or ROC are more suitable to define MCIC in their specific circumstances, the consistency of ROC and MCS values across studies may help them to use these results in practice (Tables 3 and 4) [8,9,16]. The upside of using the MCS value instead of ROC is that patients with scores showing an improvement above its value have a 95% chance of having improved meaningfully. However, in general, ROC might be more suitable, since scores from patients both reporting and denying improvement are used to calculate it, and it tends to weigh equally false-positive and false-negative misclassifications [16]. As it has been suggested, the choice between the two methods may also depend on the type of intervention or the clinical consequences of being a "false positive" or "false negative" [16]. For instance, some researchers may prefer to anticipate a difference generally corresponding to ROC (e.g. 1.5 PI-NRS points) for sample calculations in clinical trials vs. placebo, since ROC represents "the cut-off point that best discriminates between those patients feeling and not feeling that they have improved" and, since its size is smaller than MCS, it leads to larger samples. On the contrary, some clinicians may prefer to disregard differences smaller than MCS (e.g., 4 PI-NRS points) when they have to select among treatments with different safety profiles or side effects for a given patient, since that value represents "the mean change above which most patients would feel they have improved".

To be included in this study, patients had to be subacute or chronic and their pain had to be ≥ 3 PI-NRS points. Therefore, it could be argued that this could limit the generalizability of results from the current study to patients having pain for less than 14 days and to those with mild pain, and future studies could estimate MCIC in them.

The positive clinical evolution of most patients in this study made it impossible to estimate MCS and MDC for worsening (Table 2), and that should be explored in further studies.

Mean duration of pain when patients entered this study was over 540 days (Table 1). During that period, they had all received many forms of treatment and many still received them during the study [22]. Since data being analyzed in this study derive from post-marketing surveillance of neuroreflexotherapy, all of them received that specific form of treatment [22-27]. No study has assessed the potential influence of any specific form of treatment on MDC or MCIC and many studies include patients receiving heterogeneous treatments, since they are participating in randomized controlled trials [8,16]. In fact, MDC and MCIC calculations rely on patients' self-assessment of their own evolution and scores from instruments used to assess evolution of symptoms, no matter what treatments are potentially influencing that evolution. Therefore, the generalizability of results from this study are not affected by the fact that these results derive from the post-marketing surveillance of a particular form of treatment. The consistency of results from this study with those from previous reports on neck pain and low back pain patients further supports their generalizability.

In fact, using post-marketing surveillance methods in a National Health Service to assess MCIC has a number of advantages. It makes it possible to assess MCIC values in routine practice conditions, as opposed to using data from randomized controlled trials in which Hawthorne and other unspecific effects might influence patients' perception of global improvement and, therefore, the results. In addition, post-marketing surveillance makes it possible to recruit large representative samples and to minimize losses to follow-up, therefore giving a better general picture of what MCIC values are likely to be in "normal" clinical conditions.

Since no validated instruments to assess neck pain-related disability were available for Spanish speaking patients when this study started, pain was the only symptom that could be assessed and MCIC values for that variable were the only ones to be calculated. Based on previous studies in neck and low back pain patients, it is very likely that disability also influences patients' perception of general improvement [1-9,13,14,18,19], and further studies should explore MCIC for that variable in this cultural environment. However, since MCIC for pain and disability are calculated separately [1-9], the impossibility of assessing disability does not affect the validity of the MCIC values for pain deriving from this study.

The use of patients' classification of their own general clinical evolution as the external criterion is somewhat controversial, since it requires for them to compare their initial and final states [7,8]. However, that is how patients assess their own evolution in routine clinical practice. It also happens to be the usual "gold standard" for "patients' subjective global improvement" in the research setting [8], and has a high face validity. In routine clinical practice it is of the utmost importance, since it would not make sense to classify a patient as improved or deteriorated against the patient's own personal assessment [8]. For these reasons this was considered to be an appropriate external criterion for the study.

In this study, patients' own classification was rated on a 4-point scale ("completely recovered", "improved", "unchanged" or "worsened"), [30] while other studies have used 5 or 7-point scales to that end, in which "improvement" and "worsening" were split into further categories, such as "much improved", "slightly improved", "slightly worsened" or "much worse" [1-8,11]. However, those categories must usually be collapsed at the analysis phase, so it is up to researchers to decide how to group them. On the contrary, we preferred for patients to rate their own evolution in the categories that were going to be analyzed. This may have led to patients who perceived an improvement as clinically irrelevant selecting the "unchanged" category. Taking into account the objectives of this study, we find that to be suitable.

## Conclusion

The results of this study provide a reference for what "relevant" improvement means in terms of neck and referred pain for subacute and chronic neck pain patients treated in routine clinical practice. The values provided here may be useful for clinicians to interpret patients' evolution depending on the baseline severity of their symptoms and duration of pain. They can also assist researchers in selecting an MCIC value depending on the context in which it is going to be used.

## Competing interests

The author(s) declare that they have no competing interests.

## Authors' contributions

FK conceived the study and participated in its design, coordination and the drafting of the manuscript.

VA, AR, AC, AM and JZ performed the statistical analysis and participated in the drafting of the manuscript. VA also participated in the design of the study.

JC, LA, MT and MAM collaborated in coordination and data collection.

MTG, MG and NM participated in the study design, and drafting and editing of the manuscript.

All authors revised the design, revised the draft manuscript and read and approved its final version.

This manuscript does not contain information about medical device(s).

This study was funded by the Kovacs Foundation.

The authors received no individual funding for their work.

Th funding institution played no role in the study.

No benefits in any form have been or will be received from a commercial party related directly or indirectly to the subject of this manuscript.

## Pre-publication history

The pre-publication history for this paper can be accessed here:


